# Chaperone Like Attributes of Biogenic Fluorescent Gold Nanoparticles: Potential to Alleviate Toxicity Induced by Intermediate State Fibrils Against Neuroblastoma Cells

**DOI:** 10.3389/fchem.2019.00787

**Published:** 2019-11-19

**Authors:** Anzar Abdul Mujeeb, Khan Farheen Badre Alam, Ansam Wadia Faid Alshameri, Fauzia Jamal, Saba Farheen, Mohd Kashif, Anees Ahmed, Irfan Ahmad Ghazi, Mohammad Owais

**Affiliations:** ^1^Interdisciplinary Biotechnology Unit, Aligarh Muslim University, Aligarh, India; ^2^CSIR-National Botanical Research Institute, Lucknow, India; ^3^Department of Plant Sciences, School of Life Sciences, University of Hyderabad, Hyderabad, India

**Keywords:** gold nanoparticles, biogenic nanoparticles, chaperone, amyloid, fibril

## Abstract

In general, neurodegenerative disorders have a great deal of correlation with the misfolded as well as aggregated forms of protein-based macromolecules. Among various species formed during the aggregation process, protein oligomers have been classified as most toxic entities against several types of living cells. A series of chemicals have been developed to inhibit protein aggregation as a measure to regulate neurodegenerative diseases. Recently, various classes of nanoparticles have also been reported to inhibit protein aggregation. In the present study, we synthesized fluorescent gold nanoparticles (B-AuNPs) employing *Olax scandens* leaf extract. Next, an *in vitro* study was performed to assess the effect of as-synthesized B-AuNPs on the aggregation behavior of the ovalbumin (OVA) and other related model proteins. We performed an extensive study to elucidate anti-amyloidogenic properties of nano-sized entities and established that small-sized B-AuNPs manifest chaperone potential against protein aggregation. Further, we exploited as-synthesized B-AuNPs as a mean to prevent protein aggregation mediated toxicity in neuroblastoma cells.

## Introduction

Amyloidal protein toxicity has a strong implication in several neurodegenerative disorders such as Alzheimer's, Parkinson's (PD), Huntington's, and Prion disease, etc. (Chung et al., 2018). Fibrillation has been considered as a centric aggregation process that involves loss of native conformation, unfolding and inappropriate refolding of protein-based macromolecules (Chiti and Dobson, 2017). In general, the protein aggregation encompasses formation of oligomeric and other intermediate supramolecular entities, which subsequently associate to form insoluble fibrils comprising of unbranched β-sheet rich fibrillar structure (Rahimi et al., 2008). Under normal physiological conditions, the aggregation-prone residues of a native protein remain buried deep in the hydrophobic core (Beerten et al., 2012). Surface exposure of β-strand forming short amino acid residues ensues in the formation of protein aggregates (Wei et al., 2017). Various subspecies of an aggregated protein, formed during fibrillation, often manifest toxic properties against living cells (Tuite and Melki, 2007). In fact, the as-synthesized fibrillar aggregates may induce changes in the structure and function of neuronal cells and evoke apoptotic signals (Stefani and Rigacci, 2013). This eventually results in synaptic dysfunction and neurodegeneration that adversely affect the nervous system of the involved individual (Marttinen et al., 2015).

A number of small-sized molecules, which can inhibit protein aggregation process have been developed recently. In general, such molecules have the potential to inhibit aggregation by interacting directly with the precursor protein as well as various intermediates generated during the fibrillation process (Heller et al., 2017). Interestingly, some of them ameliorate aggregation process under physiological conditions (Eisele et al., 2015). Such “chaperones” mimicking molecules generally stabilize protein “native” structure, thereby prevent its misfolding (Naik et al., 2012). Moreover, other class of anti-fibrillar molecules may inhibit/reduce secondary nucleation presumably by binding to the proto fibril surface, and eventually inhibit protein fibrillation (Kazmi et al., 2018).

Nanosized metal particles with modified chemical and physical attributes have been utilized excessively in medical and other related fields (Yang et al., 2011). Recent trend, of using plant or microbe based cell lysate in the assembly of metal nanoparticles, has made great impact in the field of nanotechnology (Singh et al., 2008).

An attractive alternative approach to regulate protein aggregation process is to use artificial chaperone, in the form of nanoparticles, to inhibit the synthesis of amyloidogenic fibril structures (Young et al., 2017). This seems to be an interesting strategy to regulate protein fibrillation employing various types of nanoparticles. It is speculated that the nature of the nanoparticle, the sequence of a given protein and the ratio between nanoparticles as well as the protein are crucial in regulating the inhibition of protein aggregation (Saptarshi et al., 2013). Beside the above described factors, physical attributes of the incubation mixture such as pH, temperature, shaking, and ionic strengths may also affect the potential of NPs to regulate the protein aggregation process (He et al., 2008).

In the present study, we intend to elucidate the exact mechanism involved in B-AuNPs mediated inhibition of protein aggregation. First, we explored green synthesis of B-AuNPs employing *Olax scandens* leaf extract. The as-synthesized B-AuNPs were characterized for their intrinsic properties. Next, we studied the effect of small-sized B-AuNPs (range 10–50 nm) on aggregation of model proteins using dye binding, CD polarimetry, and fluorometric techniques. Lastly, we studied the effect of as-synthesized B-AuNPs on the generation of aggregate associated toxic species and their potential to inhibit neuroblastoma (SH-SY5Y) cells.

## Materials and Methodology

### Materials

#### Chemicals and Reagents

Ovalbumin, HAuCl_4_, Congo Red, and Thioflavin T used in the experiments were obtained from Sigma-Aldrich (St. Louis, MO) otherwise specified. Human neuroblastoma cell line (SH-SY5Y) was acquired from the American Type Culture Collection (ATCC, USA). The plant *Olax scandens* belongs to family Olacaceae is commonly known as parrot O*lax*. The plant is a shrubby climber in nature, generally grows in tropical countries including India. The fresh leaves from healthy plants were washed 3 times with distilled water followed by further washing with sterile Millipore water and finally dried in shade.

### Methanol (MeOH) Extract of *Olax scandens* Leaf

The shade dried *Olax scandens* leaves (50 g) were powdered followed by extraction of its chemical constituents with MeOH using Soxhlet apparatus. The dark green crude extract was subjected to evaporation on water bath to get dry mass. Next, a stock solution was made by adding 10 mg of crude leaf extract in 1 ml DMSO: H_2_O (20: 80; v/v) (Naik et al., 2015).

### Green Synthesis of B-AuNPs

B-AuNPs were synthesized by *Olax scandens* leaf extract mediated reduction of gold precursor, chloroauric acid (HAuCl_4_). The synthesis was executed by incubation of a constant volume of chloroauric acid (150 μL of HAuCl4) with varying volumes of the OS stock (150 to 500 μL). The final volume of the reaction mixture was adjusted upto 5 mL with water. The reaction mixture was continuously stirred at room temperature up to stipulated time period to obtain as-synthesized B-AuNPs. The undesirable plant products/materials were removed by centrifuging the sample at 10,000 g for 10 min. The pellet was dried at 80°C to get as-synthesized B-AuNPs.

### Characterization of B-AuNPs

#### UV-VIS Spectroscopy

UV-VIS absorption spectra were analyzed to study the biogenic reduction of gold ions to the colloidal nanostructures (B-AuNPs). The kinetics of B-AuNPs synthesis was followed by UV scanning of incubation mixture at various time points. Next, the effect of concentration of *Olax scandens* extract on the synthesis of B-AuNPs was also studied by UV scanning of incubation mixture.

#### FTIR Spectroscopy

As-synthesized B-AuNPs were analyzed by Fourier transform Infra-red spectroscopy (IR affinity-1, Shimadzu, Japan). As-synthesized B-AuNPs sample disc was co-prepared along with KBr crystals as a beam splitter. The IR study was performed to determine the presence of residual biological moieties (biomolecules from *Olax scandens* extract) in the as-synthesized B-AuNPs.

#### Transmission Electron Microscopy (TEM)

TEM was employed to study the size and shape of as-synthesized B-AuNPs at an accelerating voltage of 200 kV using JEOL transmission electron microscope. A 200-mesh copper grid, which was covered by the carbon-stabilized formvar film was used for probing the as-synthesized B-AuNPs. Uranyl acetate (2%w/v) was used as negative stain. The excess fluid was removed before TEM analysis of the sample.

#### Size Distribution Determination by Dynamic Light Scattering (DLS)

The Dynamic Light Scattering measurement (using Dynopro-Tc-04 instrument; Protein Solution, Wyatt Technology, Santa Barbara, CA) was done to determine average size and distribution of the as-synthesized B-AuNPs. The scattered light intensity was detected at 90° of the incident beam. Briefly, PB buffer [PB pH 7.4] was used to re-suspend the lyophilized powder. The solution obtained after passing through a 0.22 μm filter (Millipore) was subjected to various size determining measurements. The data was analyzed in the default mode. The average value of 20 runs(done in triplicate)was considered for assessment of the size of the as-synthesized B-AuNPs.

### Effect of B-AuNPs on the Aggregation of Model Protein OVA

#### ThioflavinT (ThT) Fluorescence Studies

ThioflavinT (ThT) (C_17_H_19_ClN_2_S) dye binds to the as-synthesized β-amyloid entity in a quantitative fashion. ThT binding spectral analysis was done by using Spectro fluorophotometer (Shimadzu RF-5301, Japan). Double distilled water was used to prepare 20 μM stock solution of ThT. OVA was stirred in the presence of the varying amount of as-synthesized B-AuNPs for various time period to study the effect of presence of the later on the synthesis of β-amyloid. An excitation wavelength of 440 was used to excite the samples dispensed in quartz cuvette with path length set at 1 cm and the emission spectra recorded between 450 and 600 nm. To determine the effect of B-AuNPs on other model proteins, we repeated the same set of experiments with HSA protein.

#### The Effect of B-AuNPs in the Genesis of OVA Fibril Was Also Followed by CR Binding Assay

The effect of B-AuNPs on OVA fibrillation process was also analyzed by Congo red (CR) binding assay. The absorbance of protein-bound CR was determined by using, UV-VIS-1800, spectrophotometer (Tokyo, Japan), in 400–700 nm range by using a cuvette of fixed path length of 1 cm. The binding of CR to β-amyloid resulted in a redshift in the spectrum of the CR in dose-dependent manner. Next, we performed CR binding assay to follow effect of B-AuNPs on fibrillation process in HSA protein.

#### Far UV CD Spectra Analysis

Far-UV CD spectral analysis was used to study the alteration in the secondary structure of OVA β-amyloid. The spectrum was recorded using JASCO spectropolarimeter J-815 instrument. The instrument was calibrated with D-10-camphor sulfonic acid using Jasco Peltier type temperature controller. Analysis was carried out at 25° ± 1°C, while the scanning speed maintained at 100 nm/min with a response time of 1 s. A scan range of 190 and 250 nm was used for the study. Four scans were performed, and average was taken for the spectrum to be plotted to minimize the associated experimental errors.

#### Surface Morphological Study of As-Synthesized NPs and OVA Fibrils Employing Transmission Electron Microscopy (TEM)

The morphology of as-synthesized NPs was analyzed by using TEM. An aliquot of B-AuNPs sample (10 μl) was placed on a carbon-coated copper grid for 1 min. Distilled water was used for washing, followed by air drying. An aqueous solution of uranyl acetate 2% (w/v) was used (45 s incubation) for negative staining of the sample. The aggregated form of OVA fibrils was analyzed employing TEM microscopy. The sample was prepared in the same manner as described in previous lines.

#### Fluorescence Imaging to Study the Toxic Effect of Fibril (Formed in the Presence of As-Synthesized B-AuNPs) on Human Neuroblastoma Cells

The human neuroblastoma cell line (SH-SY5Y) cells were cultured overnight in the CO_2_ incubator (5% CO_2_, 37°C). The log phase cells were incubated with fibril formed in the presence of increasing concentration of as-synthesized B-AuNPs.

#### MTT Cell Viability Assay

The toxicity of OVA fibril on SH-SY5Y neuroblastoma cells was determined by employing MTT assay. Briefly, SH-SY5Y cells were cultured in DMEM **(10% FBS)** media to attain 70% confluency in 96-well plates. Cells were washed with HBSS followed by treatment with early-stage OVA oligomers synthesized in presence/absence of B-AuNPs for 24 h at 37°C under 5% CO_2_. Excess of OVA oligomers was removed by washing the cells. The cells were further incubated in fresh media for 24 h. For viability assay, the cells were washed prior to treatment with 20 μl of MTT solution (5 mg/mL in PBS). The cells were further incubated for 4 h at 37°C. Subsequently, the supernatant was aspirated carefully. Finally, cells were solubilized in 200 μl DMSO. The complex purple colored formazan formed was measured at 570 nm. The healthy cells without prior exposure to fibrils were used as positive control.

### Statistical Analysis

All the experiments were independently performed in triplicates, and the values were expressed as mean ± SEM. After one-way analysis of variance, data analysis was done employing Graph Pad Prism 5.01 (California, USA). *P* < 0.05; was considered statistically significant.

## Results

### Synthesis and Characterization of Gold Nanoparticles (B-AuNPs)

**Figure 1:**
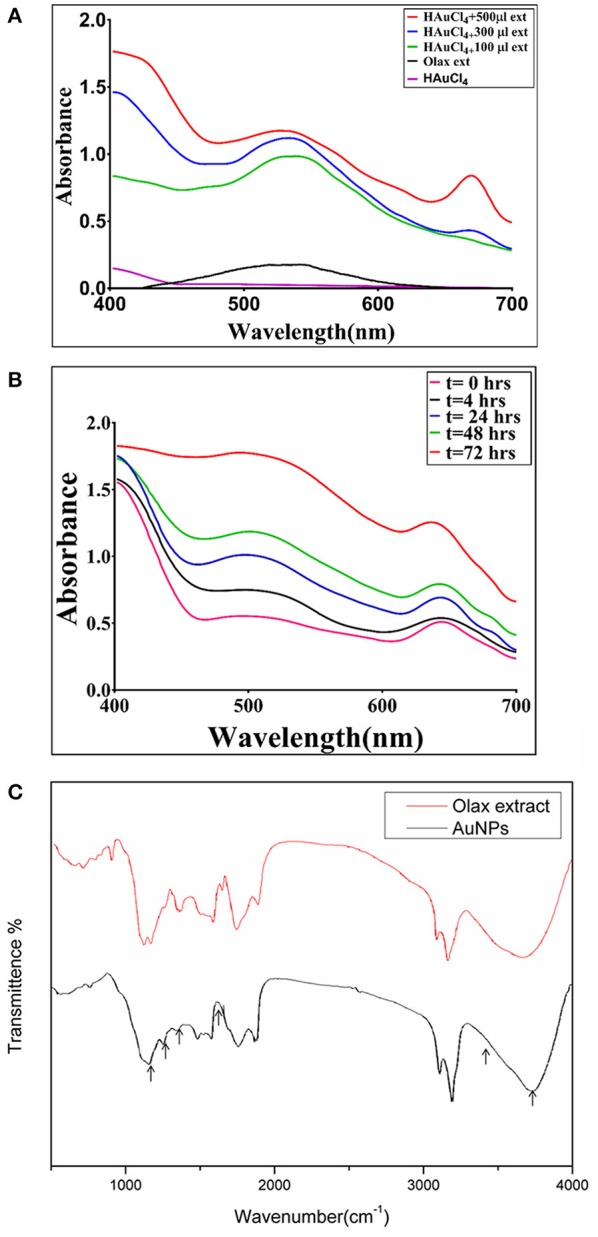
**(A)** UV–visible absorption spectrum of as-synthesized B-AuNPs fabricated with the help of *Olax scandens* leaf extract: UV-Visible spectra of gold nanoparticles formed upon incubation of HAuCl_4_ (10^−3^M) with increasing concentrations of *Olax scandens* leaf extract. The characteristic surface plasmon resonance (SPR) bands corresponding to gold nanoparticles progressively shifts toward higher wavelength with accompanying amplification in band intensity as a function of increasing amounts of O*lax scandens* leaf extract added to the incubation mixture. **(B)** Time dependent kinetics of the B-AuNPs synthesis in the presence of *Olax scandens* extract: The time dependent kinetics of gold nano-particle synthesis, as elucidated by UV scanning of the incubation mixture at various time point. The characteristic surface plasmon resonance (SPR) band of gold nanoparticles progressively increases with time, signifying the formation of gold NPs. **(C)** Representative FTIR spectrum of as-synthesized B-AuNPs: FTIR spectra of as-synthesized gold nanoparticles generated upon incubation of *Olax scandens* leaf extract with aqueous solution of HAuCl_4_. Red Curve, represenative of pure *Olax scandens* leaf extract, black curve corrresponds to as-synthesized gold nanoparticles produced upon reduction of chloroauric acid by leaf extract constituents.

The reduction of chloroauric acid to gold NPs was studied by UV–VIS absorption spectra analysis (Figure 1). The synthesis of B-AuNPs was accompanied with the formation of a cranberry color complex. No such characteristic peak was observed in the precursor HAuCl_4_ solution (Figure 1A). The incubation with increasing concentration of *Olax* extract resulted in an increase in the magnitude of absorbance with characteristic surface plasmon resonance peaks of gold nanoparticles in the range of 510–540 nm. The increasing extract content resulted in synthesis of a greater number of particles that absorbed characteristic UV-VIS electromagnetic radiation in concentration dependent manner. We observed that incubation with 500 μl (10 μg/ml stock) of the *Olax* extract resulted in maximal synthesis of B-AuNPs. Further increase in the *Olax* extract content did not induce any additional increment in the absorbance. A secondary peak at around 675 nm was also visible corresponding to florescent components present in the leaf extract. No such peak was observed when lesser leaf extract content was used in the fabrication of B-AuNPs. It seems that leaf extract residues had tendency to get adsorbed on the surface of as-synthesized B-AuNPs. It can also be inferred that characteristic fluorescent emission of the as-synthesized B-AuNPs at 675 nm is majorly controlled by adsorbed leaf extract components present on the surface of B-AuNPs as well. Or else the increased synthesis of B-AuNPs may result in to the increase in the absorbance around the 675 nm wavelength.

### Time Kinetics of the B-AuNPs Formation

The time kinetics of the nanoparticle synthesis was followed for a period of 72 h. Initially, at the 0 h time point, there was no SPR signal. At around 4 h time period, there appeared a characteristic peak in the range of 510–540 nm. Bands corresponding to surface plasmon resonance of as-synthesized B-AuNPs were observed in the visible region. For sake of clarity, we omitted depiction of band in IR region. The two SPR bands at 540 and 675 nm correspond to oscillation of electrons in the conduction band along short and long axis. The stronger band at 540 nm corresponds to transverse surface plasmon resonance of as-synthesized B-AuNPs. Whereas, the weaker band at 675 nm corresponds to electron oscillation of the alternate axis of as-formed nanostructures along with the fluorescence of the fluorescent components present in the extract.

Earlier reports suggested that *Olax* leaf constituents can also absorb at around 675 nm. We also observed a second peak at around 675 nm. This peak can be attributed to fluorescent plant content present in the incubation mixture. The appearence of more prominent can be attributed to the cumulative effect of the extract constituents along with the formation of B-AuNPs. The peak continues to grow with increasing incubation time period. Post 72 h, fully formed SPR peak of B-AuNPs was observed (Figure 1B). There was no further increase in the magnitude of absorbance beyond 72 h time point. It seems that maximum NPs synthesis was achieved at this time point.

### FTIR Spectroscopy of As-Synthesized B-AuNPs

The FTIR spectroscopy provides knowledge about presence of various functional groups in a given chemical entity. From the previous literatures it can be deduced that the various components present in the extract of *Olax* leaf such as phytochemicals and proteins may have a role in reduction of HAuCl_4_ into gold nanoparticles. Phytochemicals found adsorbed on the surface of B-AuNPs are likely to have aldehydes, ketones, polyphenolic based compounds as revealed by FTIR. It seems such phytochemicals, executed the reduction of HAuCl_4_ to AuNPs. A very strong peak corresponding to O-H stretching H-bond and phenolic groups was observed at 3,490–3,500 cm^−1^. Another peak around 1,400–1,550 cm^−1^ signified the presence of (N-H) aromatic secondary amine. It can be inferred that (N-H) stretching band was also involved in the synthesis of B-AuNPs. The region around 1,450 and 1,500 wavenumber corresponds to intermediate intensity vibrations bands of C = O and C = C. The vibrational bands of medium intensity corresponding to C = C and C = O seems to be contributed from flavonoids and terpenoids residues present in the *Olax* leaf extract. The efficient capping and stabilization of the synthesized nanoparticles can be attributed to the various leaf based phytochemicals (Figure 1C).

### TEM and DLS Analysis of the Particles

**Figure 2:**
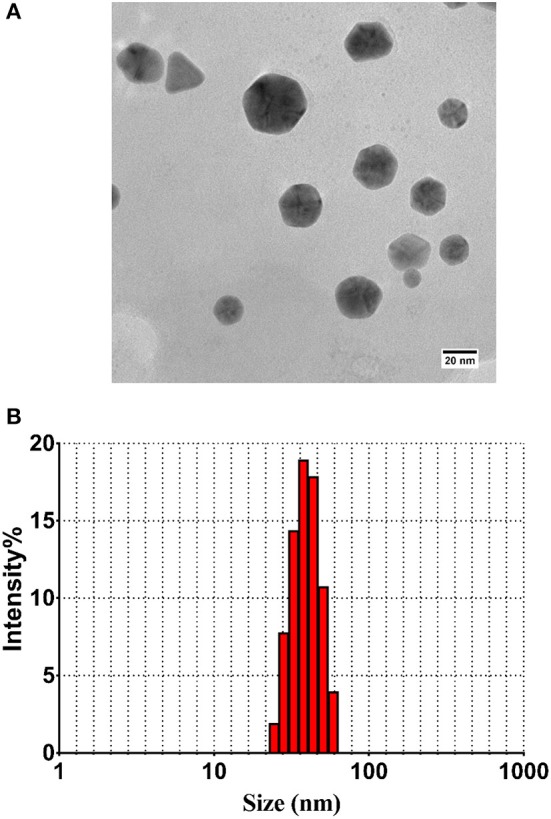
**(A)** TEM analysis depicting shape and size of as-synthesized B-AuNPs: Representative TEM image of gold nanoparticles synthesized using methanolic extract of *Olax scandens* leaf. TEM micrograph showing simultaneous presence of hexagonal and triangular nanostructures of B-AuNPs synthesized upon incubation of *Olax scandens* leaf extract with aqueous HAuCl_4_. **(B)** Size analysis of the B-AuNPs as determined by DLS analysis: Particle size analysis with DLS (Dynamic Light Scattering) suggests overall particle radii of the as-synthesized gold nanoparticles to be approximately in the range 10–50 nm.

TEM micrograph showed that as-synthesized nanoparticles consisted of well-dispersed NPs with varying shape and sizes. Majority of the as-synthesized particles were found to be of spherical to oval in shape (Figure 2A). In general, the as-synthesized B-AuNPs had a spherical and ovoid morphology ranging from 10 to 50 nm, which is consistent with DLS size measurements. The hydrodynamic diameter of as-synthesized B-AuNPs corresponds to particles suspended in liquid ambiance where average particle size was found to be greater in size as compared to size determined by TEM images (Figure 2B).

### B-AuNPs Mediated Inhibition of OVA Amyloid Synthesis

**Figure 3:**
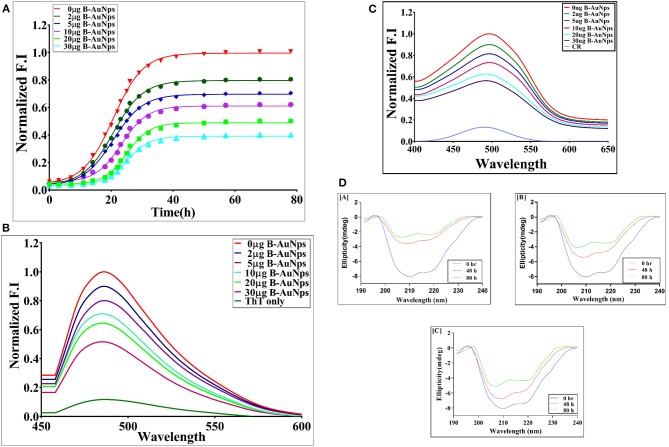
**(A)** Time kinetics of OVA aggregation in the presence of B-AuNPs: The effect of B-AuNPs on synthesis of OVA amyloid was monitored by determining ThT fluorescence associated with as-formed OVA amyloid. The OVA amyloid synthesis followed a sigmoidal kinetics that involves around 5 h lag phase before attainment of exponential phase. The presence of B-AuNPs resulted in inhibition of OVA-amyloid synthesis in concentration dependent manner. **(B)** ThT fluorescence spectra of as-synthesized OVA fibril generated in the presence of increasing concentration of B-AuNPs: Absorption fluorescence spectra of ThT bound mature OVA amyloid species generated at a given time point. The effect of presence of B-AuNPs on generation of OVA amyloid was monitored by plotting absorption fluorescence spectrum of mature OVA amyloid formed. The B-AuNPs inhibited OVA amyloid synthesis in dose dependent manner. **(C)** CR absorption spectra of as-synthesized OVA in the presence of increasing concentrations of B-AuNPs: The amyloid synthesis of OVA was induced in the presence of increasing concentration of B-AuNPs at 25°C for a definite time periods (24 h). CR solution (20 μM) in PB served as a control (CR-only spectrum). B-AuNPs were found to reinstate red shift in as formed OVA-fibril. Experimental data represent of means of three determinants ± S.D (*n* = 3). The data is representative of three different experiments with same pattern. **(D)** Far UV CD spectra of as-synthesized OVA fibril synthesized in the presence of increasing concentrations of B-AuNPs: The CD spectrum of native OVA showed characteristic α-helical structure with negative MRE peaks at 210 and 218 nm. CD spectra of OVA shaken for various time points (in the absence of B-AuNPs) showed transformation of α-helical structure to β-sheet with the increase in incubation time. The fibril formation was accompanied with alpha helix to beta sheet structural transition in the OVA protein (negative peak at 218 nm). A decrease in the β content was seen when OVA fibril formation was induced in the presence of B-AuNPs. The as-synthesized B-AuNPs inhibited OVA fibril formation in concentration dependent manner **[A]** 0 μg B-AuNPs; **[B]** 2 μg B-AuNPs; **[C]** 30 μg B-AuNPs.

ThT binding assay has been widely exploited to elucidate the possible mechanism involved in induction of protein aggregates. The solution of model protein (OVA 1 mg/ml) in fibrillation buffer (25 mM Tris buffer, 50 mM NaCl, and 1 mM DTT, 0.01% azide; pH 7) was shaken at 90 rpm for extended time period. The induction of OVA amyloid aggregate synthesis was monitored employing ThT binding assay. The ThT dye specifically interacts with crossed-β sheet structure of as-synthesized fibril, eventually resulting in a significant increase in relative fluorescence intensity of the binding complex (Figure 3A). ThT dye specific relative fluorescence intensity (RF intensity), was employed as a parameter to examine the effect of B-AuNPs on OVA fibril generation. On the other hand, as-formed fibril with exposed hydrophobic residue has greater propensity to interact with ThT. The presence of B-AuNPs inhibited OVA fibril formation in concentration dependent manner. In contrast there was greater ThT binding with as-formed OVA fibril formed in the absence of B-AuNPs (Figure 3B). Reduction in the fluorescence intensity signifies that less of the β- sheet harboring fibrils/amyloidal intermediates are being formed that in turn ensued in less ThT specific fluorescence. It can be concluded that the presence of as-synthesized B-AuNPs retards formation of the fibrils/b-sheet/amyloidal intermediates. It seems that B-AuNPs present in the reaction mixture interact with the free monomers of the protein. Infact, protein molecules in solution form a corona around the NPs reducing their availability for the formation of amyloid fibrils. There was less fibril formation when fibrillation process was induced in the presence of B-AuNPs. This in turn resulted in less ThT binding. Maximum decrease in binding was observed in the presence of 30 mg/ml B-AuNPs, suggesting that at this concentration, the inhibition of aggregation was maximum. To establish that B-AuNPs mediated inhibition of fibril formation is a universal phenomenon, we extended this inhibition study with another model protein human serum albumin (HSA). We found that B-AuNPs can inhibit HSA fibril formation with same efficiency to that of OVA fibril inhibition (Figures S1, S2).

### B-AuNPs Inhibit OVA Fibrillation in Concentration Dependent Manner as Revealed by Congo Red Binding Assay

Congo red (CR) binds to the beta-amyloid and results in red shift with enhanced absorbance (Figure 3C). The Congo red binding assay is characteristic of the protein aggregation process. The native OVA showed minimum CR absorbance around 500 nm wavelength as the dye fails to bind with native structure of OVA. The peak around 500 nm showed maximum absorption corresponding to OVA fibrillation. There was a shift of 20 nm in λmax as compared to native protein. The increasing concentration of B-AuNPs resulted in decreased CR absorbance at 500 nm. The incubation with 30 μg/ml of B-AuNPs showed absorbance similar to that of the native binding pattern of the OVA. On exceeding this concentration, the absorbance was found to be still identical to that of the native OVA. We observed that low size B-AuNPs exhibited marked aggregation inhibition at a concentration of 30 μg/ml. In general, CR binds with exposed β-sheet content present in the protein fibril. Incubation of OVA for elongated time periods in fibrillation buffer resulted in formation of β-sheet structures. This in turn results in more CR binding to as-formed fibril. Presence of B-AuNPs resulted in less fibrillation or intermediate formation and eventually minimal CR binding. Interestingly, there was less CR binding when another model protein HSA was used in place of OVA. This could be attributed to lesser fibril formation in HSA protein. Fibril formation in absence of B-AuNPs (0 μg B-AuNPs) resulted in maximal absorbance at 500 nm.

### B-AuNPs Induce Conformational Changes in the Model Protein OVA

Change in secondary structure of OVA upon fibril formation was determined by performing CD spectroscopy in far UV region (200–250). Considering the fact that fibril formation leads to change at specific wavelength, we scanned the reaction mixture at 218 nm employing CD polarimeter. The fibrillation of OVA resulted in CD spectrum of OVA with β-sheet structure (without B-AuNPs). CD spectra of OVA at 0 h post incubation with B-AuNPs showed no structural change. As shown in Figure 3D[A], native OVA, with significant alpha helical content, showed a negative peak at around 218 nm. The CD analysis suggest that fibril formation is accompanied by a structural transition in the OVA protein from alpha helix to beta sheet. The presence of B-AuNPs retarded conversion of alpha helix to beta sheet, as revealed by decrease in negative band value at around 218 nm Fibril formation resulted in characteristic CD pattern corresponding to beta-sheet rich protein shifting the curve toward a less negative band polarity. As can be seen in Figure 3D[B] there was less conversion of alpha helix to beta sheet when OVA fibrillation was executed in presence of 10 μg of B-AuNPs as compared to the fibrillation that was induced in the absence of B-AuNPs. The effect was more prominent at higher concentration (30 μg) of B-AuNPs. The presence of B-AuNPs was maximally observed at 30 μg/ml concentration of the NPs. Beyond this range the inhibition trend was similar to that of 30 μg/ml B-AuNPs (data not shown). It can be inferred that the anti-fibrillar effect of B-AuNPs followed a concentration dependant kinetics (Figure 3D).

### B-AuNPs Incur Alteration in Morphology of As-Formed OVA Aggregate as Revealed by TEM Analysis

**Figure 4:**
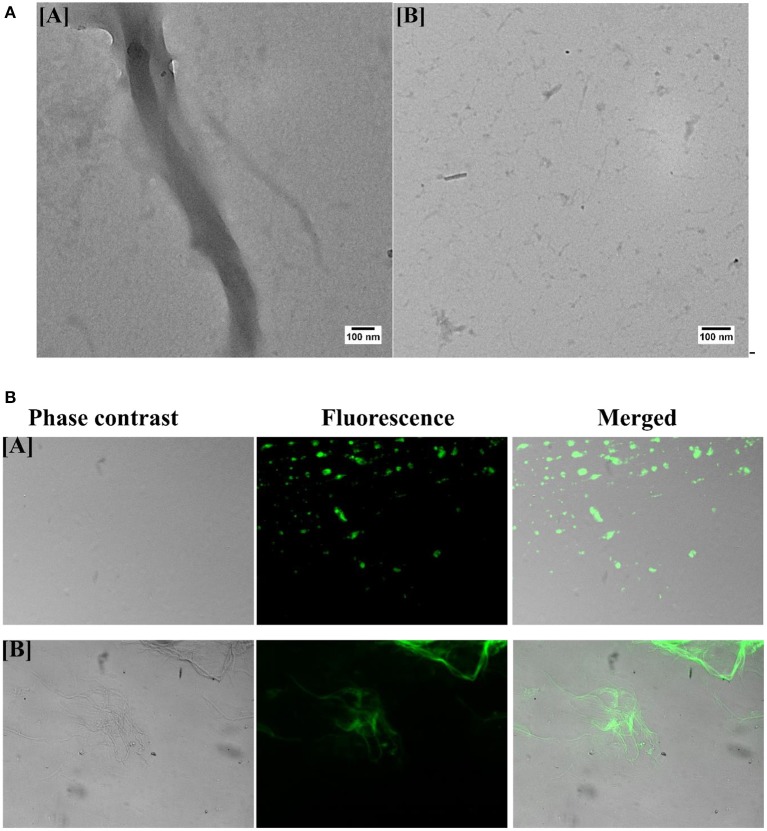
**(A)** Transmission electron microscopy of as-synthesized OVA fibril prepared in the presence of B-AuNPs: **[A]** As- synthesized OVA amyloid. **[B]** Aggregation inhibition when amyloid synthesis was executed in presence of B-AuNPs. **(B)** Green fluorescence corresponding to ThT bound as-synthesized OVA Amyloid as visualized employing epifluorescence microscopy: The epi-fluorescence micrographs of the as-synthesized OVA amyloid formed in presence **[A]** or absence **[B]** of B-AuNPs.

Finally, to analyze the effect of B-AuNPs on the aggregation of OVA and also to conform its inhibitory effect, TEM analysis of the formed aggregates was performed. Figure 4A[A] shows fully mature as-formed aggregate of OVA. It is evident that OVA-amyloid sample possessed long branched characteristic fibrillar entities. TEM image of the fibril formed in the presence of B-AuNPs revealed generation of short and sparsely populated non-fibrillar aggregates during synthesis of fibril (Figure 4A[B]).

### Green Fluorescence of OVA Amyloid Bound ThT

Fluorescence corresponding to ThT bound OVA amyloid was executed employing epifluorescence microscopy. ThT has excitation and emission spectrum peak wavelengths of approximately 450 nm/490 nm, and responsible for the observed green florescence. The epi-fluorescence pattern of the as-synthesized OVA amyloid formed in presence (Figure 4B[A]) and absence (Figure 4B[B]) of B-AuNPs had been shown in the corresponding micrographs. As evident from Figure 4B[A], there was minimal(almost negligible) fibril formation when OVA was co-incubated with B-AuNPs.

### Uptake of B-AuNPs by Neuroblastoma Cells

**Figure 5:**
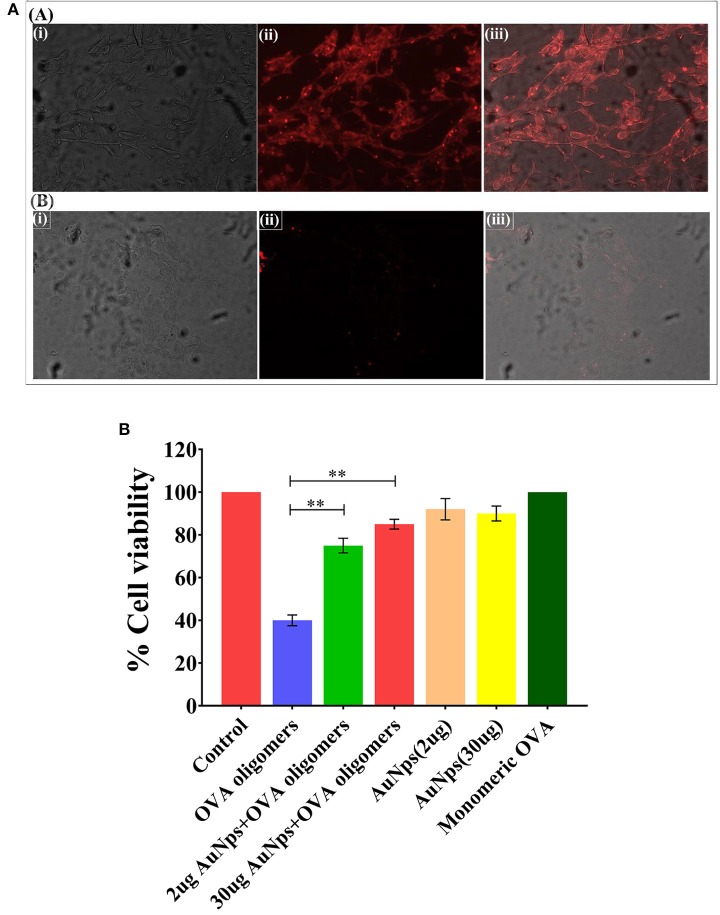
**(A)** Uptake of B-AuNPs by neuroblastoma SH-SY5Y cells: The neuroblastoma cells were cultured on the glass cover slip overnight. The incubation of B-AuNPs with SH-SY5Y cells resulted in uptake of NPs in both time and concentration dependent manner. The epifluorescence micrographs of cultured neuroblastoma cells in absence **[A]** or presence **[B]** of B-AuNPs. **(B)** MTT Cell Viability Assay: MTT cell viability assay was performed to assess potential of as-synthesized B-AuNPs to inhibit toxic OVA fibril synthesis. OVA fibril impart toxicity to neuroblastoma SH-SY5H cells, however, presence of B-AuNPs slows down synthesis of oligomeric OVA species. The less amount of toxic OVA oligomers does not adversely affect neuroblastoma cells. Significance level have been denoted with the ^**^.

The incubation of fluorescence active B-AuNPs with SH-SY5Y cells resulted in their uptake by neuroblastoma cells. The intrinsic red fluorescence corresponding to B-AuNPs suggest internalization of B-AuNPs. Once inside the cells, the B-AuNPs are likely to exert their chaperone activity (Figure 5A).

### B-AuNPs Alleviates OVA-Fibril Mediated Cytotoxicity in Neuroblastoma Cells

SH-SY5Y (human neuroblastoma cell line) cells were exposed to intermediate species of OVA fibrillation process in presence of increasing concentration of B-AuNPs. MTT assay was employed to ascertain the extent of OVA-fibril cytotoxicity. Approximately 40% death of the treated cells was caused by the OVA intermediate species. OVA caused amyloid mediated disruption of the cell membrane resulting in cell toxicity. Cell viability was increased to 75 and 83% in presence of 20 ug B-AuNPs and 30 ug B-AuNPs, respectively. The observation establishes that the anti-amyloidogenic behavior of B-AuNPs results in an increase in cell viability. For the analysis of MTT results One way ANOVA was used. (Figure 5B).

## Discussion

The interaction of nanoparticles with protein-based materials results in adsorption (corona formation) of the later on their surface (Saptarshi et al., 2013). The as-formed corona can be either of “soft type” consisting of loosely bound proteins, or “hard corona,” comprising of tightly bound protein molecules on the surface of the NPs (Van Hong Nguyen, 2017). The extent of adsorption of the corona forming protein-based materials depends on the intrinsic properties of the core nanoparticle on one hand, and the surrounding milieu on the other (Neagu et al., 2017). The adsorption of protein-based macromolecules on the surface of nanoparticles generally leads to alteration in their conformation and overall topology (Guo et al., 2014). On the other hand, the protein-based corona may also modify morphology as well as zeta potential of the core NPs (Konduru et al., 2017). Several recent reports suggest that altered aggregation behavior of NPs surface adsorbed proteins as compared to their counterpart present in the bulk solution (Radic et al., 2015). The metal NP induced misfolding of the proteins had remained an interesting criterion that has been considered crucial for NP associated toxicity in general (Tamás et al., 2018). Interestingly, the present set of data, however, establishes anti- fibrillar properties of gold NPs synthesized employing *Olax scanden*s leaf extract.

The multistep fibrillation process depends on an equilibrium that involves the subcritical, critical nuclei and monomer species of the protein involved (Lee et al., 2007). It is followed by an irreversible transition of critical nuclei into mature fibrils (Stefani and Dobson, 2003). Taking such factors into consideration it is possible to manipulate the rate of involved kinetics, initial steps involved in formation of subcritical and critical nuclei and overall whole fibrillation process in number of ways (Arosio et al., 2015). In the present set of study, we have tried to narrow down the options of the operative mechanisms involved and also to understand process of fibril formation in the presence of metal NPs. The data of the present study implicate that the nucleation step involved in OVA fibril formation is strongly disturbed in the presence B-AuNPs (Figure 3A).

The surface properties of the B-AuNPs play important role in protein perturbation during fibrillation process (Fei and Perrett, 2009). In general, it can be stipulated that monomeric OVA binds to the B-AuNPs. However, in the case of aggregation of proteins in the presence of B-AuNPs; it is difficult to discern what exact subpopulation (monomeric vs. oligomeric) of the OVA interacts with B-AuNPs. It seems that NPs can bind with various subpopulations of OVA in more than one way, therefore the binding of monomers to NPs results in the lessening of available monomers in the solution. Due to this, the equilibrium between monomer and oligomer is disturbed. Interestingly, the dissociation of as-formed aggregates leads to re-establishment of equilibrium. The genesis of critical nuclei is generally followed by their rapid growth into mature fibrils. On the other hand, the amount of sub- and near-critical oligomers may also get decreased due to their binding to NPs. This may regulate abundance of the oligomer in the bulk solution that may in turn affects the nucleation step. Interestingly, NPs bound oligomers do not undergo fibrillation. Infact, NPs binding may halt elongation process by inhibiting access of incoming monomer for its addition to protofibrils as well as intermediate state.

An important point to be noted here is that both of these mechanisms may lead to reduction in the number of oligomers in the surrounding milieu. It seems protein molecules present in the bulk solution get adsorbed onto B-AuNPs surface. This ultimately leads to decrease in the sub and critical oligomer population and also would cause blockage of the fibril synthesis pathway partially, until all the particle surfaces are saturated and new nucleus starts to form. It is likely that adsorption of monomer on the NPs surface shifts the equilibrium thereby inhibit fibrillation process. Earlier reports suggested that unfolded protein experiences a lower kinetic barrier to fibrillation, however, in the present experimental setup, the nanoparticle bound protein undergoes hindered fibrillation because of its immobilization on the particle surface (Stefani and Dobson, 2003; Arosio et al., 2015).

In general, protein-based chaperones restrict the protein aggregation mainly through two main methods namely hydrophobic shielding and proteolytic degradation (Saxena et al., 2018). In living cells, the misfolded or nascent proteins are refolded with the help of chaperones (Griesemer et al., 2014). The chaperones help in refolding of proteins through their specific interaction with the hydrophobic domain of misfolded or denatured proteins and also by encapsulating denatured proteins in their cavity or channel (Saibil, 2013). The proteins have the capacity to attain stable native conformation inside the chaperone as the environment is isolated with no forces being operative to form aggregates once inside the cavity (Saibil, 2013; Griesemer et al., 2014). The dissociation of refolded proteins from chaperones requires weak interactions between them that is considered to be critical for the functioning of chaperones.

Nanoparticles have been shown to work on the line similar to protein-based molecular chaperones (Wang et al., 2014). We hypothesize that nanoparticles interaction with protein occurs through multiple adsorption sites which can hinder the aggregation of proteins. Nanoparticles with relatively high surface area along with attachment of various different functional groups on their surface can provide binding sites to the incoming protein monomers thus can influence the growth kinetics of the protein fibrillation. The local concentration of proteins can increase because of binding of the proteins to the nanoparticles. This leads to less interaction of protein molecule with other protein molecules and eventually slows down the overall fibrillation process. Various attributes of the nanoparticles such as size, chemical nature, and other surface properties influence the kinetics of the protein fibrillation (Thanh et al., 2014).

It seems that B-AuNPs have the capacity to control the start as well as progression of fibrillation of the model protein OVA (Figure 3A). Both monomeric and intermediates of OVA formed during the aggregation can bind B-AuNPs and lead to inhibition of fibrillation process. The binding of monomer to NPs leads to their depletion that eventually ensues in shifting of equilibrium in backward direction. Also, the sub- and near-critical nuclei may bind until the surface of NPs is fully saturated thereby enforce decrease in availability of monomer species.

Next, we determined potential of B-AuNPs in successful alleviation of amyloid induced toxicity to neuroblastoma cells. We speculated that presence of B-AuNPs might reduce or lessen amyloid generation. Non-soluble β sheet oligomers formed as intermediate product during fibrillogenesis induce cellular toxicity to the living cells. B-AuNPs extend or increase lag phase of amyloid synthesis process by targeting early aggregate species thus help in delaying overall amyloid synthesis process. In other words, it can be said that toxic oligomers were seized by B-AuNPs. Once trapped, the synthesis of amyloids is reduced because of the non-availability of the intermediates. B-AuNPs mediated inhibition of Amyloid β fibril formation may have great implication in alleviation of Alzheimer's and other neurodegenerative diseases.

The B-AuNPs mediated depletion of toxic fibril species impart less toxicity to the neuroblastoma cells. Further, the incubation of B-AuNPs with neuroblastoma cells resulted in their cellular uptake as revealed by presence of fluorescing B-AuNPs particles inside the cells (Figure 5A). Once inside, B-AuNPs may exert chaperone role to regulate misfolding of protein in the neuroblastoma cells. The MTT data explicitly suggests anti fibril property of the B-AuNPs. The nanoparticles inhibit synthesis of toxic oligomers in dose dependent manner thereby regulate death of the treated cells (Figure 5B).

It can be argued that the prevailing conditions in natural systems are far more complex with plethora of proteins and other surrounding situations to interact with the protein under study and such conditions can't be provided *in vitro* for the model protein OVA to interact with B-AuNPs. However, the present study can be a stepping stone for the future pathways in the development of an effective strategy against protein associated amyloid formation and also for deciphering role of metal nanoparticles as a potent inhibitor of amyloid/fibril synthesis.

## Data Availability Statement

The datasets generated for this study are available on request to the corresponding author.

## Author Contributions

AM and MO conceived and designed the experiments. AM, FJ, KA, AAh, AAl, and SF performed the experiments. AM and MK analyzed the data. MO and IG contributed the reagents, materials, and analysis tools. AM wrote the first draft of the manuscript.

### Conflict of Interest

The authors declare that the research was conducted in the absence of any commercial or financial relationships that could be construed as a potential conflict of interest.
