# Supplementary material for: Chaperone Like Attributes of Biogenic Fluorescent Gold Nanoparticles: Potential to Alleviate Toxicity Induced by Intermediate State Fibrils Against Neuroblastoma Cells
Source: Front Chem. 2019 Nov 19;7:787. doi: 10.3389/fchem.2019.00787 (PMC6878823; doi:10.3389/fchem.2019.00787)
Supplement: Supplementary file 1 [file Data_Sheet_1.PDF]

Supplementary information

Chaperone like attributes of biogenic fluorescent gold nanoparticles: potential to alleviate toxicity of intermediate state fibrils against neuroblastoma cells

Anzar Abdul Mujeeb[1], Khan Farheen Badre Alam[1], Ansam Wadia Faid Alshameri[1], Fauzia Jamal[1], Saba Farheen[1], Mohd Kashif[2], Anees Ahmad[1], Irfan Ahmad Ghazi[†3], M. Owais[1*]

[1]Interdisciplinary Biotechnology Unit, Aligarh Muslim University, Aligarh-202002, INDIA,

[2]CSIR-NBRI, Lucknow-226001

[3]School of Life Sciences, University of Hyderabad, Hyderabad-500046, INDIA

Figure S1. B-AuNPs mediated inhibition of HSA amyloid synthesis

The potential of B-AuNps to inhibit synthesis of amyloid aggregates in another model protein, HSA was monitored employing ThT binding assay. The ThT dye specifically interacts with crossed-β sheet structure of as-synthesized fibril, eventually resulting in a significant increase in relative fluorescence intensity of the binding complex [**Figure-S1**]. There was maximum ThT fluorescence in the HSA protein when fibril formation was induced in absence of B-AuNps. However, there was less ThT binding in the samples that were incubated with increasing concentration of B-AuNPs during amyloid synthesis. Maximum decrease in rate of fibril synthesis was observed in the presence of 30 mg/ml B-AuNPs, suggesting that at this concentration, the inhibition of aggregation was maximum.

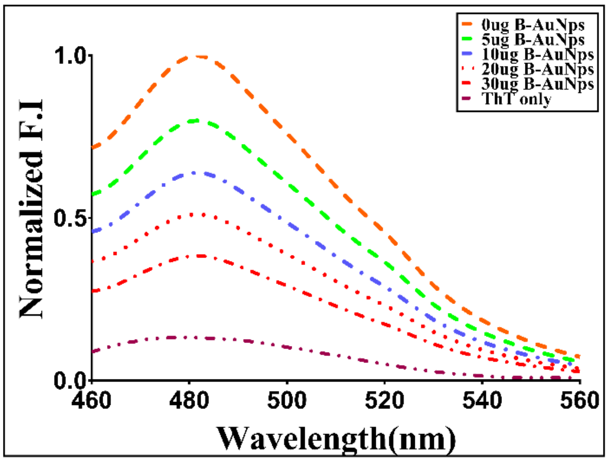

[Figure-S1]

Figure S2. B-AuNPs inhibit HSA fibrillation in concentration dependent manner as revealed by Congo Red binding assay

[**Figure-S2]**. The congo red dye binds with as-formed HSA fibril and the complex showed maximum absorption at 500 nm, with a red shift 0f around 20 nm as compared to the native protein. The increasing concentration of B-AuNPs resulted in decreased absorbance. The data established that low size B-AuNPs exhibit marked aggregation inhibition at a concentration of 30 μg/ml. The dye CR binds with the b-sheet structure of as-formed fibril. Increasing addition of B-AuNps leads to less fibrillation and less intermediate formation as revealed by CR binding pattern.

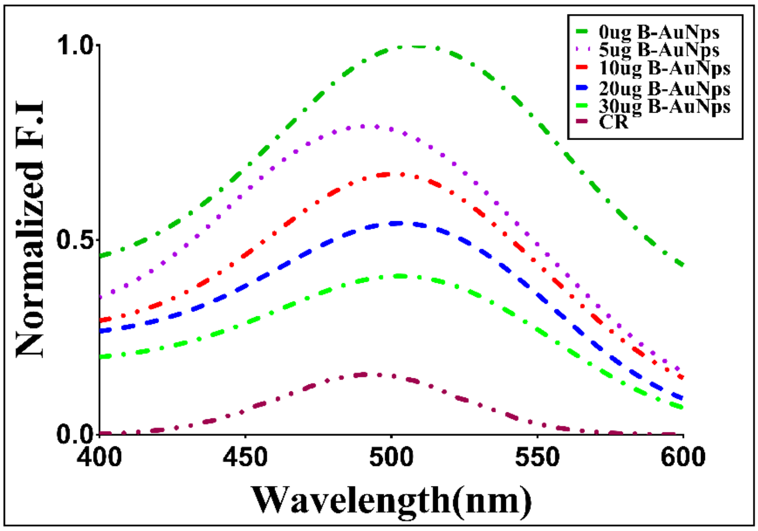

[Figure-S2]
